# Examining the ‘cosmetics placebo effect’

**DOI:** 10.1371/journal.pone.0210238

**Published:** 2019-01-10

**Authors:** Carlota Batres, Sarah S. Kramer, Caroline G. DeAngelis, Richard Russell

**Affiliations:** 1 Psychology Department, Gettysburg College, Gettysburg, Pennsylvania, United States of America; 2 Psychology Department, Franklin and Marshall College, Lancaster, Pennsylvania, United States of America; University of Toronto, Rotman School, CANADA

## Abstract

Previous studies have found a positive effect of cosmetics on certain behavioral measures, such as the tip given to waitresses by male patrons. These studies have employed confederates who usually wear cosmetics. We therefore sought to examine whether the positive effect found in these studies could, in part, be explained by a change in behavior. In order to test the possibility of a ‘cosmetics placebo effect’, we employed a confederate to solicit donations from passersby. On some days our confederate would not have any cosmetics applied to her face (i.e., no cosmetics condition), on some days cosmetics were pretended to be applied to her face (i.e., placebo cosmetics condition), and on other days cosmetics were actually applied to her face (i.e., cosmetics condition). In line with previous research, we found that across conditions men donated significantly more than women to our female solicitor, providing support for the ‘showoff hypothesis’, in which male generosity serves as a mating tactic. When investigating men’s donations in more detail, we found that the highest percentage of donations came in the cosmetics condition, followed by the placebo cosmetics condition, and then by no cosmetics condition. The effect of condition on donation rates, however, was not statistically significant. Our study was limited to one solicitor and one dependent variable (i.e., percentage of people approached who donated) and therefore future research would benefit from using more confederates as well as examining other behavioral measures. Given the influence of cosmetics use on so many real-world outcomes, we believe that further exploration into a possible ‘cosmetics placebo effect’ would be valuable.

## Introduction

Research has demonstrated that the use of cosmetics is associated with a variety of social outcomes, ranging from perceptions of physical attractiveness [1–8] to evaluations of expected job performance [2]. For example, in one study, two female confederates entered a bar either made up or not and the number of male solicitations and the latency of the first solicitation were recorded [9]. Results showed that when the confederates were wearing cosmetics, the number of solicitations was higher and the latency between the arrival of the confederates at the bar and the first solicitation was shorter. Another study used waitresses as confederates and had them wait on tables either made up or not while their tips were recorded [10]. Results showed that the waitresses received significantly higher tips from the male patrons when they were wearing cosmetics.

It is important to note that for both the bar study [9] and the restaurant study [10], confederates who usually wore cosmetics were selected. We believe that this methodological detail is of critical importance to the findings as there is a possibility that the results could, in part, be attributed to a ‘cosmetics placebo effect’. It may be possible that the confederates altered their behavior in the condition where they were not wearing cosmetics since they may have felt less confident or uncomfortable. Indeed, research has found that women report being more self-confident and sociable when they are wearing their customary cosmetics [11]. Given the influence of cosmetics on so many real-world outcomes [2, 3, 12], it is important to examine whether part of the positive effect from cosmetics use can be explained by a change in behavior.

We therefore set out to investigate the possibility of a ‘cosmetics placebo effect’ by employing three conditions: no cosmetics, placebo cosmetics, and cosmetics. The application process of the cosmetics condition was mimicked in the placebo cosmetics condition so that the confederate believed the same cosmetics were applied to her face in both conditions. This design allowed us to examine if, and to what extent, there is a placebo effect related to cosmetics use.

Testing at a bar or a restaurant, in line with the previous studies [9, 10], was not possible given the presence of mirrors, which would allow the confederate to notice she was not wearing cosmetics in the placebo condition. We therefore decided to test in an open-air location, where we had our confederate request donations from passersby. We chose to solicit donations to test our hypothesis because much research has already been devoted to understanding what influences charitable giving [13–15].

Some research has specifically examined the influence of solicitor characteristics on charitable giving [16, 17]. One study, for instance, found that men contribute more to charity when they are being observed by a woman, when compared to being observed by a man or when not being observed by anyone [16]. Given that our study used a female solicitor, we predicted that we would find a sex difference in donation rates, with a larger percentage of men, compared to women, donating when approached by our confederate.

In addition, research has shown that higher levels of physical attractiveness in female solicitors results in an increase in giving between 50–135 percent [17], with the increase being mostly driven by male donors. Previous studies have also found that cosmetics increase attractiveness [1–8], so we therefore predicted that our confederate would receive the most donations from men when she was soliciting donations wearing cosmetics. In line with the bar [9] and restaurant [10] studies, we selected a confederate who normally uses cosmetics, and since women report an increase in self-confidence and socialness when wearing their customary cosmetics [11], we predicted that the placebo cosmetics condition would receive the second highest number of donations. Lastly, without either the visual effect of cosmetics or the possible change in behavior with cosmetics use, we predicted that the no cosmetics condition would receive the least number of donations.

## Methods

### Participants

A Gettysburg College student who normally wears cosmetics was recruited to be the confederate for this study but was not informed of the goals of the study. The confederate self-identified as White and was 21 years old in the fall semester of the data collection and 22 years old in the spring semester of the data collection. The confederate approached 626 people (375 males and 251 females) to ask for donations. Ethical approval was received from the Gettysburg College Institutional Review Board, a permit allowing the solicitation of donations on Lincoln Square was issued by the Gettysburg Borough, and authorization to solicit donations for the American Red Cross was granted by a representative of the American Red Cross. Informed consent from participants could not be collected as that would alter their behavior and therefore the Gettysburg College Institutional Review Board waived informed consent under the Code of Federal Regulations, Part 46.116, because the waiver did not adversely affect the rights and welfare of the participants.

### Design

The study was scheduled for three days a week for seven weeks in the fall (collection starting at 11:30 am on Mondays, Wednesdays, and Fridays) and seven weeks in the spring (collection starting at 1:00 pm on Wednesdays, Thursdays, and Fridays). The condition (i.e., no cosmetics, placebo cosmetics, and cosmetics) was randomized across the days to avoid a weekly pattern that the confederate could deduce. Additionally, the testing day was balanced across the three conditions in order to ensure that the condition was not confounded with the day of the week. Since the donations were solicited in an open-air location, some testing days had to be cancelled due to weather. This resulted in a total of 34 usable testing days.

### Materials and procedure

The confederate would arrive at the Gettysburg College Perception Laboratory 30 minutes before commencing testing and on some days she would not have any cosmetics applied to her face (i.e., no cosmetics condition), on some days cosmetics were pretended to be applied to her face (i.e., placebo cosmetics condition), and on other days cosmetics were actually applied to her face (i.e., cosmetics condition). The confederate was told that whether or not cosmetics would be applied to her face for each testing session was randomly assigned using a coin toss. Two sets of the same brushes and applicators were used (i.e., one for the placebo cosmetics condition and one for the cosmetics condition) to mimic the same cosmetics application across conditions. For example, in the placebo cosmetics condition, a clean eye-makeup applicator and brush were used on the confederate’s eyelids but no eye-shadow was applied. On the other hand, in the cosmetics condition, eye-shadow was applied using the eye-makeup applicator and brush. In the cosmetics condition, porcelain foundation, ivory pressed powder, bronzer, pink blush, brown/gold eye-shadow, black mascara, and red lipstick were applied in every session. In the same order, the placebo cosmetics condition mimicked the cosmetics condition with the same brushes and applicators but without pigmented products. In order to mimic the feel of the cosmetics condition, a non-pigmented moisturizer, a clear mascara brush with water, and a non-pigmented lip balm were used in every session for the placebo cosmetics condition. Across both conditions, the confederate maintained her eyes closed during the application process. The process was identical across conditions and sessions.

Afterwards, the confederate and a research assistant would walk over to Lincoln Square, where the testing would take place. The confederate always styled her hair the same way and wore an American Red Cross sweatshirt. The confederate would approach every man and woman walking on their own with the following: “Hello! I’m collecting money for the American Red Cross. Do you have anything you’d like to donate?”. If she saw the same person more than once, she would not approach them a second time. Additionally, people walking in pairs or groups were not approached. Meanwhile, the research assistant (who was sitting on a bench nearby) would record the sex of the person approached and whether or not they donated. Each testing session lasted 45 minutes and all donations were collected using an American Red Cross donation box. After each testing session finished, the confederate and the research assistant would walk back to the Gettysburg College Perception Laboratory, where the confederate’s face was cleansed with makeup-removing towels on the days of the placebo cosmetics condition and the cosmetics condition. The confederate was asked to close her eyes during the cleansing so she would not see the towels.

### Placebo checks

It is important to note that prior to commencing this study, other Gettysburg College students were recruited to test whether or not pretending to apply cosmetics could be differentiated from a real cosmetics application. Three students were invited to the Gettysburg College Perception Laboratory and were told that a research assistant needed to practice her makeup application skills for a study. Each student experienced two conditions: cosmetics and placebo cosmetics (order was alternated). Afterwards, they were questioned about whether they felt any differences between the two conditions. All the students reported not feeling any differences between the two conditions and were surprised when told that in one condition cosmetics were only pretended to be applied to their faces.

Additionally, the confederate in this study was probed about what she believed the purpose of the study to be after the data collection concluded. She commented “makeup versus no makeup and seeing how much money I would get and who was more likely to donate”. She was then asked if she noticed anything across the cosmetics application days and she responded that “they all seemed the same”. When probed again about feeling any differences she commented that “it all felt the same”. When told that makeup was only applied on half of the cosmetics days and only pretended to be applied on the other half, she was surprised this was the case and commented that “it didn’t feel like that at all”. The confederate was also asked if thinking back (after knowing about the different conditions) whether she could have guessed on which days she had cosmetics on and which ones she did not and her answer was “not at all”.

## Results

A total of $566.51 was collected and donated to the American Red Cross. In the no cosmetics condition, the confederate approached a total of 208 people (128 males and 80 females). In the placebo cosmetics condition, the confederate approached a total of 210 people (130 males and 80 females). In the cosmetics condition, the confederate approached a total of 208 people (117 males and 91 females).

**Fig 1:**
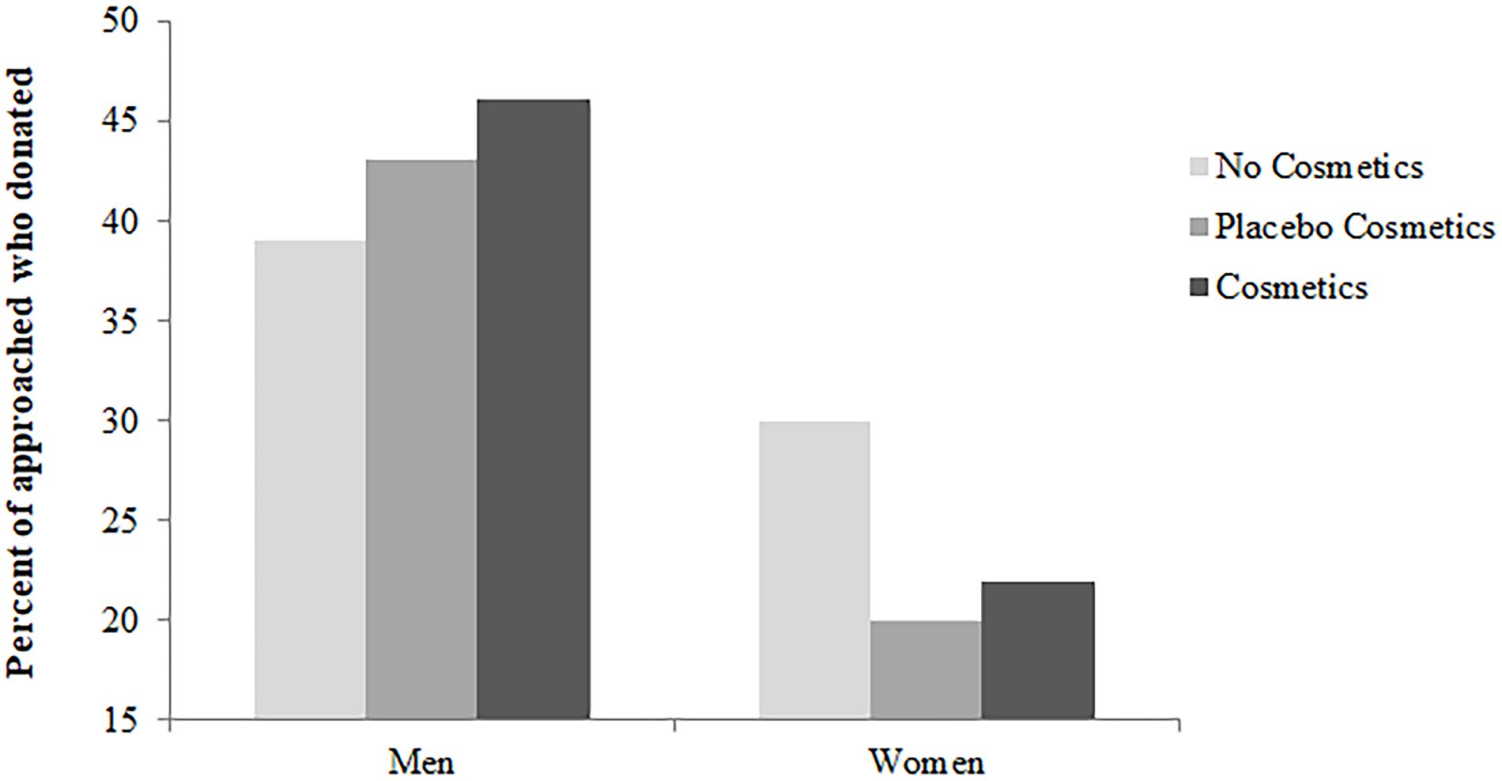
Percent of approached who donated. A comparison of the percent of men and women approached who donated across the three conditions (i.e., no cosmetics, placebo cosmetics, and cosmetics).

When averaged across conditions, 42.67% of men donated when approached. When separated by condition, 39.06% of men donated in the no cosmetics condition, 43.08% in the placebo cosmetics condition, and 46.15% in the cosmetics condition (Table 1, Fig 1). When averaged across conditions, 23.90% of women donated when approached. When separated by condition, 30.00% of women donated in the no cosmetics condition, 20.00% in the placebo cosmetics condition, and 21.98% in the cosmetics condition (Table 1, Fig 1).

**Table 1:** Frequencies of passersby approached and who donated.

	*Approached*	*Donated*
*Male Passersby*		
No Cosmetics	128	50
Placebo Cosmetics	130	56
Cosmetics	117	54
*Female Passersby*		
No Cosmetics	80	24
Placebo Cosmetics	80	16
Cosmetics	91	20

We then conducted a chi-square test between the sex of the person approached and the donation rates across conditions. There was a statistically significant association between the sex of the person and donation rates (χ(1) = 23.22, *p*<0.001). More specifically, men were more likely to donate to our confederate than women.

In order to examine whether the experimental condition (i.e., no cosmetics, placebo cosmetics, cosmetics) had a significant effect on donation rates, separate chi-square tests were run for men and women. We found no statistically significant associations between the experimental condition and donation rates for either men (χ(2) = 1.27, *p* = 0.530) or women (χ(2) = 2.49, *p* = 0.288). We then ran follow-up one-tailed chi-square tests using only the two conditions that have been examined in previous studies (i.e., no cosmetics and cosmetics [9, 10]). We again found no statistically significant associations between the experimental condition and donation rates for either men (χ(1) = 1.26, *p* = 0.161) or women (χ(1) = 1.43, *p* = 0.153). The same pattern of results was observed when conducting a binary logistic regression, with sex of the person approached contributing to the model (Wald = 22.72, *p*<0.001) and cosmetics condition not contributing to the model (Wald = 0.047, *p* = 0.829).

## Discussion

We predicted higher rates of giving when the confederate was in the cosmetics condition than the placebo condition, and more giving in the placebo condition than the no cosmetics condition. We also predicted higher rates of giving overall by men than women. We found a significant sex difference in donation rates, with a higher percentage of men approached by our female confederate donating when compared to women. Regarding the cosmetics conditions, we found that the highest percentage of donations from men came in the cosmetics condition, followed by the placebo cosmetics condition, followed in turn by the no cosmetics condition, though none of these differences were statistically significant.

Landry and colleagues [17] found that men and women donate similar amounts when the solicitor is male. When the solicitor is female, however, men donate significantly more than women. Our study replicates this result since we found that men were more likely to give a donation to our female confederate than women. This finding supports the ‘showoff hypothesis’ [18], in which men’s generosity may have evolved as a mating signal. Indeed, research has found that men contribute more to charity when they are being observed by a woman, compared to being observed by a man or not being observed by anyone [16], providing further evidence for male generosity being used as a mating tactic.

Moreover, Landry et al. [17] found that higher levels of physical attractiveness in female solicitors led to an increase in giving, with the increase being mostly driven by men. Given that cosmetics have been found to increase attractiveness [1–8], we predicted that our female confederate would receive the most donations by men when she was soliciting donations wearing cosmetics. We did find that the highest percentage of donations from men came in the cosmetics condition, however, this difference was not statistically significant.

We also predicted that our confederate would receive the second highest percentage of donations from men in the placebo cosmetics condition since women report an increase in self-confidence and socialness when wearing their customary cosmetics [11]. While we did find this to be the case for men, the difference was again not statistically significant. Lastly, we had predicted that the no cosmetics condition would receive the least number of donations since this condition would neither have the visual effect of cosmetics or the possible change in behavior with cosmetics use. We did find that the lowest percentage of donations from men came in our no cosmetics condition. This difference, however, was again not statistically significant.

While our pattern of results for men’s donations were as predicted (i.e., the highest percentage of donations came in the cosmetics condition, followed by the placebo cosmetics condition, and then by no cosmetics condition), the effect of condition was not statistically significant. We also did not find a statistically significant effect of cosmetics condition on women’s donation rates. For women, the highest percentage of donations actually came in the no cosmetics condition. Previous research has found that women are particularly harsh in their judgements of attractive women [19], and therefore, donations by female passersby may be influenced by their perception of the solicitor’s attractiveness (i.e., enhanced physical attractiveness due to cosmetics or enhanced confidence due to cosmetics or placebo cosmetics). Further research to better understand the different patterns that emerged for male and female donors would be beneficial. Future research is also needed to investigate if there is indeed a ‘cosmetics placebo effect’, where women change their behavior due to feeling less confident or uncomfortable when not wearing their customary cosmetics. If so, part of the positive effect of cosmetics on social outcomes found in the literature [9, 10] could be explained by a change in behavior.

Unlike previous studies [9, 10], we found no statistically significant difference in male behavior between the no cosmetics condition and the cosmetics condition. A power calculation, using G*Power (α = 0.05; Power = 0.80; one-tailed) and the parameters found in Jacob et al. [10] (i.e., 51% gave a tip in the makeup condition and 34% gave a tip in the no makeup condition), indicates that our sample size was sufficient to detect a cosmetics effect between those two conditions (i.e., 208 men were needed and we tested 245 men). This suggests that willingness to donate is not subject to the same cosmetics effect as that found using other behavioral measures (e.g., willingness to leave a tip [10]). For instance, in the waitress study [10], the targets had repeated interactions with the confederate before they decided on the amount of tip they would leave. In our study, the targets only had seconds before they had to decide whether or not to comply with our confederate’s request. Additionally, giving a tip is a behavior directed towards the confederate whereas giving a donation involves the confederate merely as a conduit. The confederate gains nothing from the donations being made and the identity of the charity itself could also be an influential factor. Moreover, patrons at a restaurant are prepared to pay for their meal and therefore have cash or are able to use their credit cards to leave a tip. In our study, many passersby stated they were unable to donate because they were not carrying any cash with them. Thus, future research would benefit from examining social outcomes other than soliciting donations from passersby.

Another possible reason for our null results is that the difference in perceived attractiveness may not have been large enough. Cosmetics have been found to increase attractiveness [1–8], but it would have been helpful to have collected ratings for the confederate across the conditions in order to compare the differences in attractiveness. In addition, future studies would also benefit from having more than one confederate as research has found that cosmetics have little effect on attractiveness judgments when compared to between-person variability due to identity [20] and that the increase in attractiveness due to cosmetics depends on the initial attractiveness of the wearer [21]. Further examining sex differences in donation rates [16] while employing both male and female confederates would also be beneficial. Lastly, another way to examine the possibility of a ‘cosmetics placebo effect’ would be to compare confederates who normally wear cosmetics with those who normally do not wear cosmetics.

In conclusion, we did find a sex difference in donation rates, with a higher percentage of men approached by our female confederate donating when compared to women. We did not, however, find a significant effect of our experimental condition (i.e., no cosmetics, placebo cosmetics, cosmetics) on donation rates. Given the influence of cosmetics use on so many real-world outcomes [2, 3, 22, 23], we believe that further exploration into a possible ‘cosmetics placebo effect’ would be valuable.
